# Timely referral saves the lives of mothers and newborns: Midwifery led continuum of care in marginalized teagarden communities – A qualitative case study in Bangladesh

**DOI:** 10.12688/f1000research.13605.1

**Published:** 2018-03-23

**Authors:** Animesh Biswas, Rondi Anderson, Sathyanarayanan Doraiswamy, Abu Sayeed Md. Abdullah, Nabila Purno, Fazlur Rahman, Abdul Halim

**Affiliations:** 1 Reproductive and Child Health Department, Centre for Injury Prevention and Research, Bangladesh (CIPRB), Dhaka, 1206, Bangladesh; 2 United Nations Population Fund (UNFPA), Dhaka, 1207, Bangladesh

**Keywords:** Midwives led continuum of care, marginalised teagarden communities, mothers and newborns, referral, Bangladesh

## Abstract

**Background: **Prompt and efficient identification, referral of pregnancy related complications and emergencies are key factors to the reduction of maternal and newborn morbidity and mortality. As a response to this critical need, a midwifery led continuum of reproductive health care was introduced in five teagardens in the Sylhet division, Bangladesh during 2016. Within this intervention, professional midwives provided reproductive healthcare to pregnant teagarden women in the community.  This study evaluates the effect of the referral of pregnancy related complications.

**Methods: **A qualitative case study design by reviewing records retrospectively was used to explore the effect of deploying midwives on referrals of pregnancy related complications from the selected teagardens to the referral health facilities in Moulvibazar district of the Sylhet division during 2016.  In depth analyses was also performed on 15 randomly selected cases to understand the facts behind the referral.

**Results: **Out of a total population of 450 pregnant women identified by the midwives, 72 complicated mothers were referred from the five teagardens to the facilities. 76.4% of mothers were referred to conduct delivery at facilities, and 31.1% of them were referred with the complication of prolonged labour. Other major complications were pre-eclampsia (17.8%), retention of the placenta with post-partum hemorrhage (11.1%) and premature rupture of the membrane (8.9%). About 60% of complicated mothers were referred to the primary health care centre, and among them 14% of mothers were delivered by caesarean section. 94% deliveries resulted in livebirths and only 6% were stillbirths.

**Conclusions: **This study reveals that early detection of pregnancy complications by skilled professionals and timely referral to a facility is beneficial in saving the majority of baby’s as well as mother’s lives in resource-poor teagardens with a considerable access barrier to health facilities.

## Introduction

Globally 830 maternal deaths occur every day, 99% of which occur in developing countries ^1, 2^. According to the World Health Organization, roughly 303,000 maternal deaths are caused as a result of pregnancy and childbirth related complications ^3, 4^. Globally, about 3.7 million neonatal deaths occurred within the first 28 days, with 75% in the first week of life ^5^. Only 19 out of 186 countries have achieved the Millennium Development Goal-5, related to reduction in maternal mortality ^6^; unfortunately, Bangladesh is not one of them. Estimations suggest that about 87% of maternal deaths occurred in South Asian and Sub-Saharan African regions ^7^. According to the Demographic and Health Survey, neonatal mortality rates range from 28 to 54 per 1000 live births in Bangladesh, India and Pakistan ^8^. In 2010 the Bangladesh Maternal Mortality and Health Care Survey (BMMS) claimed that the lifetime risk of maternal death is 1 in 500 due to pregnancy and delivery related complication, and two third of these deaths occurred in the postpartum period. A study in Bangladesh found that 38% of the maternal deaths occurred by haemorrhage, which is the most common cause, 20% occurred by eclampsia, and 8.1% occurred by sepsis ^9^. Another study in the teagarden area of Bangladesh revealed that maternal death in teagarden areas is higher due to lack of knowledge on maternal complication. Ignorance, traditional myths, family restriction on seeking better care, and dependency on traditional birth attendants and village doctors also influence these maternal deaths in teagarden communities ^10^.

Referral is the process of coordinated movement of health care seeker to reach a high-level care within a small window of time ^11^. The goal of timely referral is to minimize or prevent the delay for transportation (called second delay), and ensure pre-hospital care while transporting a patient to the referral facility ^12, 13^. In 2014, Directorate General of Health Services ( DGHS) reported that out of 120 maternal deaths 47 deaths occurred in the teagarden area of Moulvibazar district of Bangladesh. Estimations suggest that about 46.4% of maternal deaths occurred at home, and 7.1% while the women were on route towards a facility; this indicated the delay occurred as a result of delay in decision making of which facility to take the mothers for management, and arranging transport to go to the facility ^14^. Another study stated that 22.2% of maternal deaths occurred with more than 6 hours delay in decision-making and 12.9% of deaths occurred with 1–2 hours transportation delay ^9^. In light of this, it can be assumed that ensuring emergency obstetric care services, and quick referral during the perinatal period can help reduce maternal deaths. To safeguard the reproductive age (15–49 years) of a woman, continuous care from family and community, along with support in getting easy access to referral healthcare facilities, is needed ^15^. Transportation support, timeliness of referral, and inter-facility transfer are major contributing factors found to reduce the rate of maternal deaths ^12, 16^. A social autopsy study of maternal deaths found that very few mothers sought facility based care during complications, and that ensuring timely referral through transportation saved the lives of many of them ^14^. It is recommended that five Emergency Obstetric and Newborn Care (EmONC) services, including four basic EmONC (BEmONC) and one Comprehensive EmONC (CEmONC), should be available and geographically distributed for each 500,000 individuals of a population ^17^. The component of care (consisting of antenatal care, identification of high risk mothers, safe delivery conduction by skilled birth attendant, timely referral of complicated mothers and postnatal care including essential newborn care.) with high quality services can be ensured by the good referral system at all levels, both in facilities as well as in communities by the trained health care providers ^9^. A shifting process is developed after the identification of high-risk pregnancies from a risk based approach to provide skilled care during delivery, and emergency obstetric care when complications occur ^18, 19^. This approach is not adequate to reduce maternal and neonatal mortality as the capacity is limited at the primary level of care, and is difficult to access in the referral facilities remaining in most of the low-income countries ^20^. Professionally, a referral transport system must be managed for providing some basic intervention to the patient before reaching the referral facility ^21^.

An intervention named *“*Bagan Mayer Jonno *”* has been implemented in the selected teagardens in the Moulvibazar district. The project ran through counseling and courtyard meetings of pregnant mothers, as well as an advocacy meeting with their guardians regarding quick referral of complicated mother. This project also supported the communities in detecting high-risk mothers by the active participation of volunteer and professional midwives. It also managed the provision of transportation and assistance of volunteers to ensure a quick and safe referral procedure. The present qualitative study describes the referral system using the midwifery led service delivery in five selected teagardens of Moulvibazar district in Bangladesh.

## Methods

Qualitative method was used to collect information in this study. The referral records of 2016 in selected five teagardens were reviewed retrospectively and qualitative information of selected 15 referral cases were collected though in-depth interviews at the community. 

### Context of the tea gardens

The average distance between a teagarden and Upazila Health Complex (UHC) varies between 12- 20 kilometers. Approximately, a population of 25,000 people with around 300 pregnant mothers at any point in time live in these gardens.

### Bagan Mayer Jonno

As part of the intervention, community volunteers called ‘Bagan Sebika’ were placed in the community, and professional midwives were situated in teagarden health facilities. Bagan Sebika (Paid Volunteer) perform community based activities including home based counseling, courtyard meetings, and advocacy meetings with the pregnant mothers and family members. Bagan Sebika also facilitated the mothers recieving antenatal care (ANC) at the facility. They also accompanying the referred mothers to the referral centre. Midwives’ role at teagarden facility includes ANC, counseling, delivery, referral, and postnatal care (PNC). Midwives also conduct delivery, referral, and PNC at a community level. Midwives also supervised the activities of Bagan Sebika at the community level.

A total of 25 Bagan Sebikas worked in the five selected teagardens. They were assigned to conduct regular home visits to households and met to pregnant mothers. The Bagan Sebikas raised awareness on various issues such as birth preparedness, pregnancy complications, danger signs, and the importance of referral. If the Bagan Sebika identified any complicated or high-risk pregnancy case, they immediately communicated with the professional midwife over mobile phone. Professional midwives are usually experienced in identifying high-risk pregnancies through ANC checkups and previous medical history of the patient. Based on severity of the complication, the professional midwife along with the Bagan Sebika motivate family members of the high-risk pregnant mother to quickly refer to the higher referral centers including the UHC, district hospital and Teagarden central hospital. This counseling assists family members in being aware of the situation and the risk involved, provides them with information on where to seek care, and motivates them to make quick decisions. The Bagan Sebika also assists family members to organize transport, and assist them throughout the referral process. All these steps combined together helps decrease instances of delay in decision making [first delay] and transportation delay or second delay in the target population. In cases of severe complications, the midwives themselves might also help in organizing transportation.

***The present study***. The present study was conducted by a facility-based retrospective record review of all referral cases occurring at the referral hospital from the selected five teagardens from January to December 2016. According to 2016 records, a total of 72 high risk pregnant mothers were referred from these five teagardens to the referral centres (Upazila health complex, district hospital and teagarden central hospital). Each teagarden has both permanent workers (registered) and causal workers (unregistered). The teagarden authority provides referral support for the registered mothers (workers), whereas, for the unregistered mothers, the referral support is very low or absent.

The professional midwives used a structured tool to document the referral history and treatment at the teagarden facilities and did follow up all referral mothers until outcome at the referral facility though Bagan Sebika. [ Table 1].

**Table 1.** Information of five teagardens selected for the study.

Name of the sub-district	Name of the teagarden	Population	Distance from UHC (Km)	Distance from district hospital (Km)	Type of facilities	Referral centre	No. of referral cases in 2016
Sreemongol	Amrailchara	4641	20	45	Hospital	Central teagarden hospital (Balisara Medical hospital), upazila health complex, Moulvibazar District Hospital, Medical college Hospital, Sylhet	19
	Rajghat	6394	12	32	Hospital		18
	Khejurichara	5171	11	31	Hospital		5
Kamalganj	Mirtinga	6378	10	17	Hospital	District Hospital, Moulvibazar	19
	Phulbari	2876	3.5	25	Dispensary	Upazila health complex, Kamalganj	11

To conduct the retrospective record review of referral centers, a structured tool was developed by the research team. The tool contained data on mother’s particulars, current pregnancy history, antenatal care, complications, treatment history, referral details, preparedness of the facilities to manage emergency obstetric complications and delivery outcome. This review was carried out by the professional midwives working in the teagarden facilities. The record review included socio-demographics of the mother, medical condition of the referred mother, causes of referral, and view of the feedback of the referred mother and their family members [ Table 2].

**Table 2.** Process of collecting information for case series studies.

Process	Types of information collection	
Record review	• Profession of Referred mother • Age of the Mother • Gravida of the referred mother • Period of Referral • Time of referral	• Place of referral • Process of delivery of referred mother • Outcome of referral • Cause of referral
Case stories analysis	• Support of the intervention • Description of remarkable referral cases	

***Data collection***. A total of 72 referral case data were entered into SPSS software (version 24.0). After entry, all data was checked for missing data and consistency. Once checking was complete, the data was cleaned, and all analysis was done using software SPSS. For case scenario description, a total of 20% of cases (n=15) were purposively selected from the five teagardens (three cases from each garden). Midwives went to the household and organized a meeting for each of the cases. The Midwife invited the family members, relative and neighbours to the meeting to gather on responses from the family and community, as well as understand the referral linkage and service delivery in the facility. The Bagan Sebika in the community organized the meeting based on suitable date and time given by the community. Descriptive statistics were computed for all variables of interest. Frequencies were established to examine the demography of referred mothers, condition of mothers during referral, and documented causes of referral. The project support and remarkable findings of the cases were analyzed through review of the case stories collected from the teagarden facilities. Themes were identified after reading and re-reading of the case stories ^22, 23^ and finally thematic analysis was performed.

### Ethics and consent

This study under “Bagan Mayer Jonno” intervention has been approved by the national ethical review committee of CIPRB (memo- CIPRB/ERC/2016/010). Verbal and written consent were received from each of the referred mothers before collecting the information for the study. 

## Results

**Figure 1.**
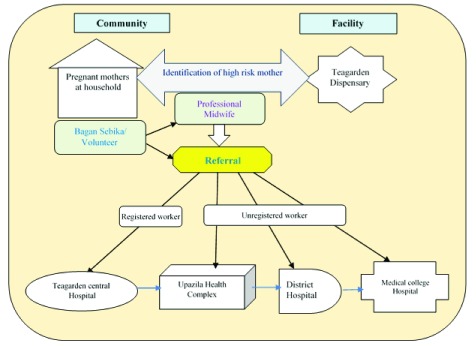
Referral process from the selected five teagardens to the higher referral centre.

From the review of records from 2016, Bagan Sebika identified the complicated mothers and immediately informed the project midwife. Then the project midwife decided whether the case needed to be referred. The project midwife also identified mothers as high risk during their routine ANC for referral. A total number of 72 complicated pregnancies (16%) were identified from a total of 450 pregnant mothers. These complicated mothers were identified at different stages during their antenatal visit, or during delivery, or immediately after delivery. Mothers informed the Bagan Sebika if any complication arose. Bagan Sebika also identified complicated mothers during their regular household visit. Then Bagan Sebika immediately informed to the project midwife. Professional midwives ensured immediate referral to the higher center after consultation, and coordinated with garden midwives, doctors and Bagan authorities. Unregistered workers in all cases directly referred to the Upazila or District facility, whereas registered workers were taken immediately to the garden’s existing referral system. In about 85% of cases, the transportation support was provided for referral of the complicated mothers, and of them in 75% of cases the Bagan Sebika (Volunteer) participated during referral of the mothers [ Figure 1].

### Age and occupation of the mothers

The referred mothers were mostly young. About 44% of mothers referred were in the age group 17–20 years, whereas 18% and 38% of mothers were from the age group of 21–25 years and 26–35 years, respectively. About 16.7% of referred mothers were housewives and the remaining were from other professions. Highest percentage (51.4%) of referral was among the unregistered teagarden workers (mothers), whereas only over 11% was registered teagarden workers. [ Table 3].

**Table 3.** Referred mothers’ characteristics.

Characteristics	Number	Percentages
Age of the mothers 17–20 Years 21–25 Years 26–35 years	32 13 27	44.4 18.1 37.5
Gravidity 1st Gravida 2nd Gravida 3rd Gravida 4th Gravida	28 25 14 5	38.9 34.7 19.4 6.9
Occupation of the mothers Registered teagarden Worker Unregistered teagarden Worker Housewife Others (includes school teachers)	8 37 12 15	11.1 51.4 16.7 20.8
Period of referral During Pregnancy During Delivery After Delivery	9 55 8	12.5 76.4 11.1
Referred from Mother’s home Teagarden dispensary	52 20	72.3 27.7
When referred 6 am- <10 am 10 am- 2 pm 2 pm- 8:30 pm	20 28 22	27.8 38.9 33.3
Mode of delivery of referred mothers Normal Vaginal Delivery (NVD) Caesarean section (CS)	62 10	86.1 13.9
Delivery outcome of referred mothers Livebirth Stillbirth	68 4	94.4 5.6

### Gravida and stage of the referred mother

39%, 54% and 7% of referred mothers were identified as 1 ^st^ gravida, 2 ^nd^ to 3 ^rd^ gravida and 4 ^th^ gravida. Most of the mothers referred were in the labour stage (76%), whereas 12.5% were referred during the pregnancy period, and 11.1% after the delivery conduction [ Table 3].

### Place and time of referral

**Figure 2.**
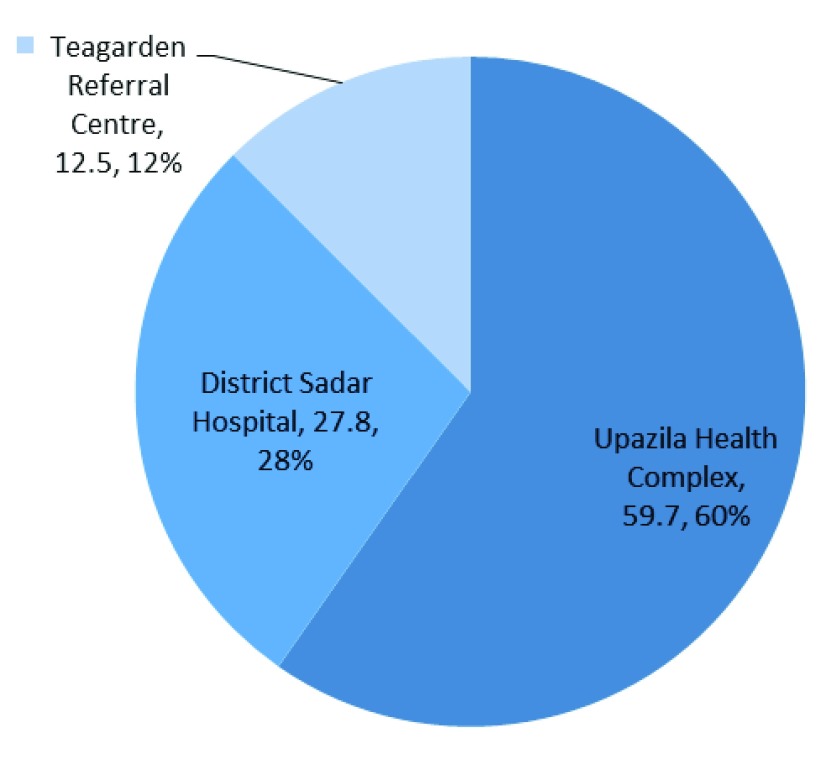
Place of referral of complicated women.

With project support, about 60% mothers were referred to Upazila Health Complex and 28% referred to Sadar district hospital. Only 13% of registered mothers or dependent workers of the teagardens were referred to teagarden referral center [ Figure 2]. The time range at which most of the mothers (about 42%) were referred was between 10 a.m. to 2 p.m., where usually doctors, nurses and midwives are available in the government facilities. The remaining referrals occurred at times when only nurses and midwives are available in the facilities. But about 28% and 30% of mothers were referred within the period of 6 a.m. to before 10 a.m., and after 2 p.m. to 8:30 p.m. which is the vital period when doctors or service providers may not be found at government facilities [ Table 3].

### Mode of delivery and outcome of referred mother

About 14% of referred mothers needed Caesarian section for complications and 86% were normal vaginal delivery conducted by a nurse or midwife in the referral center. 94% of mothers delivered livebirths and 6% delivered stillbirths (2) and intrauterine deaths (2) at referral facilities with the assistance of skilled health care providers [ Table 3].

### Cause of referral

**Figure 3.**
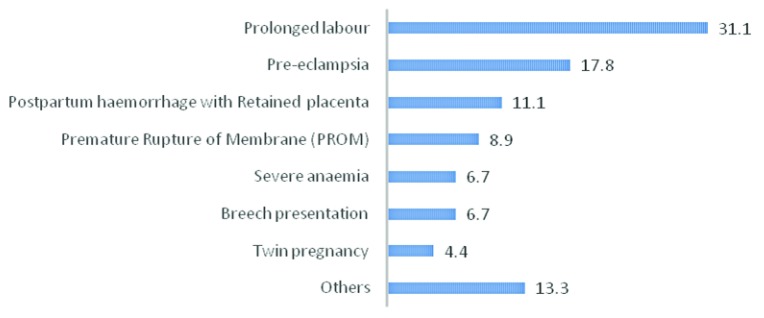
Distribution of referred mothers by cause of referral.

Most frequent causes for referral were due to prolonged labour (31%) and after that pre-eclampsia (about 18%). Moreover, another cause of referral found were retained placenta with post-partum haemorrhage, premature rupture of membrane, severe anaemia, breech presentation, twin pregnancy and others (~11%, ~9%, ~7%, ~7%, ~4% and ~13% respectively) [ Figure 3].

### Delay to start treatment at referral center after complication arises

**Figure 4.**
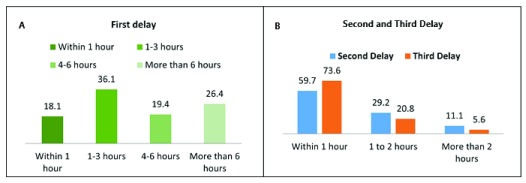
Analysis of the three delays reported for referral of complicated mothers. ( **A**) First (decision) delay- delay indecision to seek care. ( **B**) Second (transportation) delay- delay in reaching to the facility, Third (treatment) delay- delay in receiving treatment.

The delay includes first (decision), second (transportation) and third (treatment) delays, which started from the complication arising, up to receiving treatment. In about 46% of cases family members needed more than 4 hours to make a decision as whether to seek care at a facility or not. Whereas about 60% cases reached from teagarden dispensary to the referral center (UHC) within one hour, and 74% cases women received treatment within one hour after arriving at the facility. Midwifery counseling as well as transportation support from the project influenced much in reducing the community delays mainly first and second delay [ Figure 4].

### Case scenario description

A total number of 15 cases were selected randomly out of 72 cases for in-depth analysis and case scenario description. These description includes the socio-demography of the referred mothers, condition of the mothers for referral, responses of the family members and society, referral linkage and services delivery at referral centre [ Table 4].

**Table 4.** Case Scenario description of the selected 15 referral cases.

Case Number	Key scenario	What happened	Response in the family & Society	Referral linkage	Service delivery at facility
Case-1	A 30 years old woman at 4 ^th^gravida, 9 months pregnant, lives in Laltila Basti. She is a permanent worker & her home is, 23 Km away from the referral centre.	The volunteer visited the mother’s home & informed the midwife about her condition. She felt severe abdominal pain from the eighth month. At her 9th month of pregnancy she suffered from dysentery and gradually became weak.	The previous three babies were delivered at home by an untrained attendant. The family planned for conducting the delivery at home. *“I visited four times at the garden* *dispensary & received 7 iron* *tablet each time. I couldn’t easily* *go to the hospital during my* *complication due to the distance* *& lack of vehicle from my home.”-* Mother said	After complication, the mother was carried to garden hospital with the suggestion of the volunteer and Panchayat (committee consisting of 12–15 community leaders in a teagarden). The decision delay was 10.5 hrs. Volunteers carried the mother to garden hospital by CNG (Compressed Natural Gas) vehicle. Midwife confirmed the complication & referred the mother from garden dispensary to garden central hospital. Volunteer assisted to carry her to the central hospital by the garden car.	After three days of admission the mother delivered a livebirth normally at the garden central hospital with the assistance of a nurse. *“It would be difficult to save* *the mother’s life if there was* *further delay to come to the* *hospital”-* nurse of the referral centre said
Case-2	35 years old pregnant mother at 3rd Gravida. Married before 15 years. The couple are un-registered garden workers.	She suffered from severe anaemia. At 9 month of pregnancy a sudden ruptured membrane occurred. They called a traditional birth attendant for delivery. Gradually her condition became worse with no progression of labour.	The traditional birth attendant who lived to next village (TBA) tried for delivery for a long time. About one & half days passed after her labour pain & first stage become prolonged. *“No need to go hospital for* * delivery. I can assist the delivery* *at home.”-*TBA said The brother in law of the mother informed the Volunteer after about 2 days of complication. .	The volunteer motivated the family member for quick referral & carried her to UHC. Decision delay was 7 hours. The mother delivered a live baby in CNG when they reached close to health complex but the complication started with retention of placenta. Pregnant mother said *" I was very* *weak when my labor started, that is* *why I could not give much pressure.* *The volunteer advised my family* *member to bring me to hospital. I* *delivered my baby at CNG"*	At UHC the umbilicus was cut with septic measurement. After 2 hours when placenta was not removed then they referred the patient to Moulvibazar district Hospital. The patient reached at Moulvibazar Dist. Hospital accordingly and the placenta was removed there with proper management.
Case-3	26 years old 36-week pregnant woman of unregistered worker at 2 ^nd^gravida.	Prolonged labour for about 14 hours. *"this mother is an* *unconscientious mother. She* *didn't come for ANC during* *pregnancy.”-* Midwife said	The family members didn’t recognize the complication. Family members delay care seeking. Spiritual and cultural beliefs made them delay more	The mother reached the referral centre after 6 hours of complication had started at home. *“The volunteer* *motivated the family member* *for quick referral to facility from* *community."-* Panchayat member said	Volunteer carried the mother to UHC. Due to critical condition the mother was referred to District hospital from UHC & mother delivered a livebirth there with the assistance of nurse.
Case-4	25 year old unregistered pregnant woman at 1 ^st^gravida. The mother was at nine month of pregnancy.	The mother had high blood pressure with breech presentation of the baby.	The family member ignored the complication of mother. But panchayat member motivated them for referral. *“if we could not be informed* *about the condition of this mother* *by the volunteer at the proper* *time, the mother couldn’t be* *referred”. -* Panchayat said	Husband of mother informed volunteer about labor pain started at home. The volunteer informed the midwife about the condition of mother. Decision delay was about 3 hours. Volunteer carried the mother from home to garden hospital. The midwife referred the mother to UHC. Transport delay was more than ten hour. The mother further referred to district hospital. Volunteer assisted the family to go to hospital by CNG.	The family member decided to admit mother in a private clinic due to the critical condition of mother & baby. Then doctor conducted the delivery by C-section
Case-5	28 years old pregnant woman at 2 ^nd^gravida with 9 month of pregnancy received 4 ANC from teagarden dispensary. She was an unregistered worker.	The mother was identified as high risk during ANC as the complication of breech presentation, twin pregnancy and pre-eclampsia	The Traditional birth attendant of that community participated as attendant of this pregnant mother with the suggestion of volunteer. *“We did not have any money* *in our hand and as I am* *unregistered worker, the garden* *will not provide anything for me.”-* husband of the mother said	Volunteer of the garden identified mother with complication during her regular home visit. Detailed information of mother was collected previously by the volunteer. Then the volunteer immediately communicated with midwife & midwife came and identified her as high risk mother & referred to UHC.	The mother delivered livebirth by C-section in UHC conducted by the doctor of the facility
Case-6	A 25-year-old unregistered pregnant worker at 2 ^nd^gravida lived in teagarden. Her husband is also a casual worker.	During 30 weeks of her pregnancy her labour pain started. After three days of labour pain she informed family members. Deteriorating mothers condition	Their financial condition was so poor that’s why her husband could not bring her in hospital. Her condition getting worse and her life and the baby's life was in danger.	After 3 days of labour pain they informed the Volunteer. After counseling with the husband and the other family member and assurance of covering of transportation cost he allowed her to be brought to the UHC.	The mother delivered normally at UHC with the assistance of nurse. *My son may not survive if I* *stay at home and delivery was* *performed at home”-* mother said
Case-7	17 years old non-worker pregnant woman at 9 month of pregnancy with 1 ^st^gravida. Her husband is an unregistered worker.	At 36 week of pregnancy the membrane was ruptured. She also had breech presentation of the baby.	The mother received 4 ANC from bagan dispensary provided by midwife. She wanted to conduct delivery at facility but father in law did not	*“I had labour pain for many hours,* *Midwife came to my home and she* *found that it’s not possible at home.* *She said to immediately go to UHC.”-* Mother said	The mother delivered normally a livebirth in UHC with the assistance of a doctor
Case-8	18 years old non- worker mother at 1 ^st^gravida with 9 month of pregnancy lived in garden. Her husband was a casual worker.	Prolonged labour with more than 15 hours.	The family member decided to conduct delivery at home by TBA. Lack of transport, distance and travel time to reach health facilities, lack of appropriately trained staff and negative attitudes of health workers.	Then the volunteer communicated with the midwife. They motivated the family member for quick reference of this mother to UHC & said about the transport cost support. Then mother was carried to UHC by CNG.	C-section delivery conducted by the doctor in UHC. Doctor of the referral centre said *"It is really a complicated* *case. If the mother had not* *arrived on time then the life of* *mother & neonates would have* *been in danger."*
Case-9	20 years old pregnant housewife at 1 ^st^gravida with 9 month of pregnancy.	The mother suffered from anaemia. After delivery at garden hospital the neonate was suffered by birth asphyxia	Complication was not recognized as seriously by the family members. Family members delay care seeking Spiritual or cultural beliefs may reinforce delay	Volunteer carried the mother from home to garden hospital for delivery conduction. After delivery, the midwife referred the mother & neonate for complication to UHC.	Mother delivered at garden hospital by midwife and then referred to UHC; after 2 days of treatment from UHC again referred to district hospital. after 1 day treatment from district hospital the mother & neonates returned home safely
Case-10	19 years old un- registered worker pregnant woman at 3 ^rd^gravida. Her husband was also an un-registered worker	Midwife conducted the delivery at home but placenta was not removed. The mother was referred for Retained placenta	The family member wanted to conduct delivery at home due to their family tradition *"This mother didn't come at* *facility for ANC during pregnancy.* *Her husband was also ignorant* *on MNH care at facility”-* Midwife mentioned	Guardians informed volunteer after 12 hrs of labour pain & she informed to midwife. Midwife conducted delivery at home but when placenta was not removed she carried the mother immediately to district hospital.	After admission in district hospital the placenta was removed by doctor & nurses. The mother then safely returned home after one day observation.
Case-11	28-year-old non-worker pregnant woman at 3 ^rd^gravida.	The mother received 2 ANC during pregnancy. She had the complication with preeclampsia, severe head pain & weakness	Family member had negative attitudes about the behavior of health worker. *“I couldn’t* *talk properly about my last* *menstruation period which made* *it difficult to proper provide EDD* *calculation. I even didn’t follow* *the advise of Bagan Sebika and* *Midwife didi and didn’t inform of* *my delivery pain on time. So, I* *had to face lots of problem.”-* The referred mother mentioned	Midwife identified the mother at high risk & carried her to sadar hospital, and admit, getting medicine support from social welfare office, routinely follow-up.	After getting proper treatment the mother safely returned to home
Case-12	19 years old pregnant mother at first gravida. She was an unregistered worker.	Severe pre-eclampsia during pregnancy. *“I didn’t recognize that my wife* *had such complications. She* *developed swelling of legs and* *face. Our new Didi working in* *garden identified the problem* *and immediate carried my wife* *to District Sadar Hospital.”* Husband of the mother said	Mother mentioned that during pregnancy she visited only two times in hospital. I didn’t indicate the importance of going to the hospital for checkups.	The mother referred for headache & blurred vision to district hospital. The midwife carried the mother to referral centre. Doctor conducted the checkup & suggested to take medicine properly.	*"Doctors said that the patient* *condition is not good. Patient* *condition got worse due to* *severe anaemia and said to* *arrange blood. Midwife didi* *arranged the blood to save* *my wife’s life".* Husband of the mother said
Case-13	A non-registered worker of 29 years of age was referred from the teagarden at her 4 ^th^ gravitas lived in teagarden.	The mother complication includes severe anaemia, edema and preeclampsia. At 9 month of pregnancy the mother had prolonged labour & placenta previa	The family member first carried traditional birth attendant after labour pain. When she failed then after 15 hrs they communicated with volunteer. *“I had no money to transfer my* *wife. New Didi ensured me that* *transportation cost will be given.* *The volunteer went with my wife.”-* Husband said	The volunteer identified the complicated mother & immediately communicated with Midwife. Then the midwife came and advise to refer the mother immediately after examination. She also motivated the family member for taking quick decision of referral. Volunteer immediately communicated with the CNG driver and participated with the mother during referral and stay with her up to safe referral to home	*“My child was safely delivered* *after two days hospital stay. If* *I didn’t get such support, my* *wife’s and child life might have* *been under threat”-* husband said
Case-14	20 years old registered worker at 2 ^nd^gravida	The mother received 4 ANC from teagarden dispensary provided by midwife. At 9 month of pregnancy she had high blood pressure with Antepartum haemorrhage and trace Urine Albumin (2+) & previous history of PPH & prolonged labour.	Guardians of mother informed midwife & volunteer immediately at labour pain started. Midwife referred her to UHC Kamalganj due to complication. Panchayat president was accompanied with mother during referral.	The family members were concern to carry the mother at UHC immediately after referral. The nurse & doctor provided special care of the mother at UHC. After 6 hrs after admission the mother delivered normally a live birth with the assistance of nurse.	*Continuous motivation* *of Midwife didi with the* *transportation support made* *my family quickly decide to go* *to the facility. I am very much* *thankful to this project for its* *support.”-* mother said
Case-15	20 years old pregnant mother at 1 ^st^gravida is an unregistered worker in the garden	The mother received 3 ANC from garden dispensary provided by midwife. At her 8 month of pregnancy she had the complication of membrane rupture and fluid discharge.	Family member immediately communicated with volunteer after complication arises as they informed previously.	Midwife went to mother’s home after getting information & referred the mother to district hospital for complication. Volunteer carried the mother to district hospital.	Ultrasonogram was conducted & admitted in district hospital. The mother delivered a live birth by C-section with assistance of doctor & nurse.

## Discussions

The study revealed that among the referred mothers around 51% were unregistered workers who referred with the support of the project Bagan Mayer Jonno as they were not entitled to get any referral support from teagarden authorities. About 76% of mothers were referred during the period of delivery and 31% referred with the complication of prolonged labour. Most of the mothers (about 60%) were referred to the Upazila Health Complex and after referral about 14% mothers delivered by Caesarian-section at the facilities. A study conducted in rural Tanzania showed that about 28% of pregnant women were referred from primary level of care to tertiary level to ensure their better pregnancy outcome.

The same study also concluded that the most common referral complications found were multiparity (35%), young age of mother (30%), obstetric complications mostly due to prior history of caesarean section (12%), and previous existed prenatal risks like high blood pressure, severe anaemia etc. (12%) ^20^. On the other hand, our study found that 31% of mothers referred with prolonged labour, 18% with pre-eclampsia, 11% with post-partum haemorrhage (PPH) due to retained placenta, 9% with premature rupture of membrane (PROM), 18% with severe anaemia, breech presentation & twin pregnancy, and remaining 13% with other complications.

Proper transportation with cost support along with a good communication technology is the prime concerns in establishing an effective referral ^17^. Our study is also consistent with the findings that almost all referral occurs with transportation support, along with extra assistance from a midwife or volunteer, ensure the lives of many vulnerable mothers. The counseling of the midwife about the severe condition of the mother, as well as its dreadful consequences, and assistance of volunteers during referral motivated the family to quickly make their decision on referral.

Our study showed that about 50% of referred mothers received treatment within 6 hours of referral and 10.6% within 2 hours. Addressing of second delay, or transportation delay, has a significant role in reducing maternal mortalities. Many studies showed that referral transportation should be available within 30 minutes of worsening condition of a mother, so that the complicated mother can be taken to a referral center as early as possible to initiate her treatment ^24^. A mechanism needs to be established for the proper utilization of easily accessible functional transport services, which could be either from government, or from a private referral transport services ^24, 25^. Our study found that availability of transport support and assistance of volunteers from that teagarden enhanced quick referral, which consequently reduced the first and second delay.

This study found that quality ANC support by a midwife from respective gardens not only helped to identify high-risk mothers, but also further assisted the family to make a decision and prepare to delivery at a facility. Projections show that the Government of Bangladesh (GoB) already started midwifery education in all nursing institutes from 2012 and the GoB have the mandate to continue this midwifery led service delivery system until 2021, with the vision to serve hard to reach communities of the country. Another study revealed that to ensure basic and life-saving intervention to the patient, consistent support of a skilled staff should be available until the patient reaches referral facilities. However, several studies stated that it is difficult to pre-determine complication occurrence during pregnancy or childbirth ^17, 24^. The government mandate to continue the midwifery led service delivery until 2021, it is therefore necessary to regularly review the referral indicators and counsel on complication readiness, as well as birth planning by a health attendant to improve compliance on maternal referral ^20^.

## Conclusions

Early detection of complicated mothers and quick transfer to the referral center can ensure the survival of many mothers and neonates. The GoB has plans to scale up the unique midwife led service delivery (both basic and emergency health care services) system to support high-risk mothers of under privileged communities including the teagardens. The teagarden board, owners of the teagardens and local government, including policy makers of every level, must come forward to work together in finding out the best possible way to support the mothers of teagarden. At the community level, professional midwives play a key role in timely referral of a complicated mother to the facility. An integrated approach based on existing government health care delivery system with support from garden health facilities for timely referral of complicated mothers can be beneficial in reducing maternal and neonatal mortality in Bangladesh which in turn will be effective in reaching sustainable developmental goal on time.

## Data availability

Data is stored at the CIPRB. Due to sensitivity of the data (contains identifying information), permission is required from the Ethical Review Committee (ERC) of CIPRB, Dhaka, Bangladesh for sharing data with a third party. Data can be requested from the CIPRB, who will contact the Ethical Committee to gain approval to share the data. The conditions for gaining data access are a formal request with a clear objective and formal permission from the Ethical Committee. Please contact the corresponding author in order to request the data though email at info@ciprb.org.

## References

[1] World Health Organization (WHO): Global Strategy for Women’s, Children’s and Adolescents’ Health, 2016–2030.New York: United Nations;2015 Reference Source

[2] World Health Organization (WHO): Maternal Mortality. *Fact Sheet.*Media Centre;2016 Reference Source

[3] World Health Organization (WHO): True magnitude of stillbirths and maternal and neonatal deaths underreported.Media Centre,2016 Reference Source

[4] World Health Organization (WHO): Why do so many women still die in pregnancy or childbirth?2015 Reference Source

[5] Lawn JE Cousens S Zupan J: 4 million neonatal deaths: when? Where? Why? *Lancet.* 2005;365(9462):891–900. 10.1016/S0140-6736(05)71048-5 15752534

[6] World Health Organization (WHO), United Nations Children's Fund (UNICEF), United Nations Population Fund (UNFPA), *et al.*: Trends in maternal mortality: 1990 to 2015.2015 Reference Source

[7] World Health Organization (WHO): Knowledge Summary: Women’s & Children’s Health. *Progress towards MDGs 4 and 5.* Reference Source

[8] Oestergaard MZ Inoue M Yoshida S: Neonatal mortality levels for 193 countries in 2009 with trends since 1990: A systematic analysis of progress, projections, and priorities. *PLoS Med.* 2011;8(8):e1001080. 10.1371/journal.pmed.1001080 21918640 PMC3168874

[9] Halim A Utz B Biswas A: Cause of and contributing factors to maternal deaths; a cross-sectional study using verbal autopsy in four districts in Bangladesh. *BJOG.* 2014;121 Suppl 4:86–94. 10.1111/1471-0528.13010 25236640

[10] Biswas A Dalal K Abdullah AS: Maternal complications in a geographically challenging and hard to reach district of Bangladesh: a qualitative study [version 1; referees: 2 approved]. *F1000Res.* 2016;5:2417. 10.12688/f1000research.9445.1 27853517 PMC5089125

[11] Giovine A Ostrowski C: Technical report on Improving Transportation and Referral for Maternal Health: Knowledge Gaps and Recommendations. *Wilson Cent.* 2010 Reference Source

[12] Raj SS Manthri S Sahoo PK: Emergency Referral Transport for Maternal Complication: Lessons from the Community Based Maternal Death Audits in Unnao District, Uttar Pradesh, India. *Int J Health Policy Manag.* 2015;4(2):99–106. 10.15171/ijhpm.2015.14 25674573 PMC4322633

[13] Potluri P: Emergency Services in India: Counting on betterment. *Asian Hosp Healthc Manag.* 2009 Reference Source

[14] Biswas A Halim MA Dalal K: Exploration of social factors associated to maternal deaths due to haemorrhage and convulsions: Analysis of 28 social autopsies in rural Bangladesh. *BMC Health Serv Res.* 2016;16(1):659. 10.1186/s12913-016-1912-6 27846877 PMC5111193

[15] Alva S Wang W Koblinsky M: The Continuum of Care for Maternal and Newborn Health in South Asia: Determining the Gap and its Implications.Washington DC,2011;1–6. Reference Source

[16] Singh S Doyle P Campbell OM: Transport of pregnant women and obstetric emergencies in India: an analysis of the ‘108’ ambulance service system data. *BMC Pregnancy Childbirth.* 2016;16(1):318. 10.1186/s12884-016-1113-7 27769197 PMC5073462

[17] World Health Organization (WHO), United Nations Population Fund (UNFPA), Children’s Rights and Emergency Relief Organization (UNICEF), *et al.*: Monitoring emergency obstetric care: a handbook.Geneva WHO,2009 Reference Source

[18] Campbell OM Graham WJ, Lancet Maternal Survival Series steering group: Strategies for reducing maternal mortality: getting on with what works. *Lancet.* 2006;368(9543):1284–99. 10.1016/S0140-6736(06)69381-1 17027735

[19] Ronsmans C Graham WJ, Lancet Maternal Survival Series steering group: Maternal mortality: who, when, where, and why. *Lancet.* 2006;368(9542):1189–200. 10.1016/S0140-6736(06)69380-X 17011946

[20] Pembe AB Carlstedt A Urassa DP: Effectiveness of maternal referral system in a rural setting: a case study from Rufiji district, Tanzania. *BMC Health Serv Res.* 2010;10(1):326. 10.1186/1472-6963-10-326 21129178 PMC3003655

[21] Murray SF Pearson SC: Maternity referral systems in developing countries: current knowledge and future research needs. *Soc Sci Med.* 2006;62(9):2205–15. 10.1016/j.socscimed.2005.10.025 16330139

[22] Irving S: Interviewing as Qualitative Research - A Guide for Researchers in Education and the Social Sciences.Teach Coll Columbia Univ USA,2006 Reference Source

[23] Boyatzis RE: Transforming qualitative information: Thematic analysis and code development.Sage;1998 Reference Source

[24] Mavalankar DV Rosenfield A: Maternal mortality in resource-poor settings: policy barriers to care. *Am J Public Health.* 2005;95(2):200–203. 10.2105/AJPH.2003.036715 15671450 PMC1449152

[25] Adsul N Kar M: Study of rogi kalyan samitis in strengthening health systems under national rural health mission, district pune, maharashtra. *Indian J Community Med.* 2013;38(4):223–228. 10.4103/0970-0218.120157 24302823 PMC3831692

