# Supplementary material for: Timely referral saves the lives of mothers and newborns: Midwifery led continuum of care in marginalized teagarden communities – A qualitative case study in Bangladesh
Source: F1000Res. 2018 Mar 23;7:365. [Version 1] doi: 10.12688/f1000research.13605.1 (PMC5887077; doi:10.12688/f1000research.13605.1)
Supplement: Peer review history

# Peer review: Referee response for version 1

**DOI:** 10.5256/f1000research.14779.r32400

**Published:** 2018-04-05

**Authors:** Munia Islam

**Affiliations:** 1 Save the Children (Bangladesh), Dhaka, Bangladesh

- The article is very well articulated and the findings are supporting the discussion and conclusion very strongly. Additionally, it gives deep insight and shares positive experience of a community based referral intervention in the area of maternal health to a pro-poor, marginalized and isolated community. However, minor language editing will make the article more scientific, lucid, authentic and reader friendly. For example, the author could use the word ‘explore’ instead of ‘evaluate’ in the last line of the introduction part in the abstract.
- Methodology in abstract part needs reorganizing and rephrasing where qualitative method is described. The introduction of the methodology section could commence like this-

o   “This case study is designed as a mixed-methods retrospective assessment to explore the………”

 The body of the method section in the abstract can be described in this way,

 o   “In-depth interviews and retrospective document were carried out to …… Thematic analysis was performed to analyze the qualitative data.”

- The Bangla phrase, “ *Bagan Mayer Jonno*” should be mentioned in English “garden for mothers”) for the non-Bengali speakers e.g.
- Method section in the main article needs to be elaborated and re-organized to make it reader friendly and self- explanatory. This section needs revision to maintain cohesion and coherence.  
- Other than midwife is there any provision of additional service providers in the center inside the tea garden?
- Do midwives conduct home delivery?
- How do they maintain referral record? How is it documented?
- What does mean by the term ‘professional midwife’?
- Data processing procedure is described under ‘data collection’ sub-heading. It should be renamed as ‘data analysis’ or ‘data processing’. This section needs to be revised to address the cohesion and coherence also.
- Using the term, ‘case study’ instead of ‘case story’ will shape it more scientific.
- Need elaboration in the data analysis of qualitative methods. Need to mention, especially what types of qualitative method are used here. Sometimes, ‘case story’ and sometimes ‘IDI’s were mentioned, but the reader may feel difficulty to understand.
- Case scenario description’ part may go to the method section. Otherwise, you need to rephrase the sub-heading such as ‘findings from the case studies’ or rewrite the body of the paragraph in line with the previous sub-heading, so that it could be understood that you are describing result, not the process or method.
- The study reveals many opportunities to reduce the maternal mortality and morbidity of tea garden mothers. However, does this study uncover any challenges or obstacles that need further attention? Additionally, one of the recommendations should be ‘the scale-up of this intervention as a model for other marginalized communities in remote areas, who are experiencing ‘poor’ maternal health services.

I have read this submission. I believe that I have an appropriate level of expertise to confirm that it is of an acceptable scientific standard.

# Peer review: Referee response for version 1

**DOI:** 10.5256/f1000research.14779.r32399

**Published:** 2018-04-03

**Authors:** Edwin van Teijlingen

**Affiliations:** 1 Centre for Midwifery, Maternal & Perinatal Health, Faculty of Health & Social Sciences, Bournemouth University, Bournemouth, UK

Timely referral saves the lives of mothers and newborns: Midwifery led continuum of care in marginalized teagarden communities – A qualitative case study in Bangladesh

 This is an interesting article on the workings of midwifery, especially referral by midwives, in a district in Bangladesh. The case study approach is appropriate but the description of the Methods is slightly odd. This is a typical case study based on secondary analysis. But the authors do not mention ‘secondary analysis’, let alone give a reference to a methods paper /textbook on the topic. Most record studies are retrospective, i.e. researchers using the record data after it was written.  “A qualitative case study design by reviewing records retrospectively...”

 Also it is possible that authors used a Content Analysis (Krippendorff 2004) rather than a ‘general thematic analysis’?

 The Discussion needs a section on the Strengths & Limitations of this particular way of using Secondary Analysis in a Case-Study Approach. Any maternity record has incomplete data, unclear recordings, etc. None of this mentioned in the text. 

 Abstract

 The expression “76.4% of mothers were referred to conduct delivery at facilities,” is not quite right ‘women perhaps don’t conduct deliveries, you can say women deliver, or women give birth

 I read the Abstract and wondered why randomly selected in the sentence: “In depth analyses was also performed on 15 randomly selected cases to understand the facts behind the referral.”  I would have expected purposively selected case, namely ones that highlight particular aspects of the case the authors would want to highlight/stress. But when I came to page 4 the authors state that the 15 cases are purposively selected. BUT on page 7 of 15 the authors repeat the Abstract “A total number of 15 cases were selected randomly out of 72” This needs to corrected.

 Introduction

 Perhaps the reader needs a little bit more information about the state of midwifery in the country. In Bangladesh the three-years diploma curriculum following global ICM standards was introduced in 2010 (Bogren *et al*. 2015). It introduced a six-months post-basic advanced midwifery programme for graduate nurses. So what was the training of the midwives in this study? Where they post 2010 qualified or where some midwives trained prior to this date?

 Grammar, style & typos

 The authors use a mixture of American and British English. I would have preferred British English. They mix words like ‘labour’ (=British English) and in the Abstract ‘hemorrhage’ (=US English) and in the main text on page 3 ‘haemorrhage’(=British English)

 In the title (and elsewhere in article) I would use a hyphen in ‘  … newborns: Midwifery led continuum of …’ with a hyphen, i.e.: ‘Timely … and newborns: Midwifery-led continuum of …’

 Similarly in the Abstract and throughout I would have expected a hyphen in: “pregnancy related complications…”  to read: “pregnancy-related complications…”  Also page 3 “few mothers sought facility based care during” should be “…sought facility-based care …”.

 It is perhaps ugly to start a sentence with a number, there are two cases in the Abstract “the facilities. 76.4% of..” AND “section. 94% deliveries…”

 In Abstract I think plural is needed in the sentence “majority of baby’s as well as mother’s lives” to read: “majority of babies’ as well as mothers’ lives….”

 In the Abstract I think in the word ‘only’ in the sentence “  .. and only 6% were stillbirths…”  is a judgement by the authors perhaps not shared by the women/families who had a still birth. Remove the word ‘only’!

 Page 3 of 15 there are word glued together, e.g. “maternaldeaths”

 You could argue that all mothers are complicated. In the Abstract you should state something like“72 mothers with pregnancy-related complications” instead of  “72 complicated mothers”

 Remove capital in “The Midwife…” page 4 of 15.

**References:**

 Krippendorff, K. (2004) Content Analysis. An Introduction to its Methodology, London: SAGE

 Bogren, M.U., Wigert, H., Edgren, L., Berg, M (2015) Towards a midwifery profession in Bangladesh – a systems approach for a complex world, *BMC Pregnancy & Childbirth *15:325

I have read this submission. I believe that I have an appropriate level of expertise to confirm that it is of an acceptable scientific standard, however I have significant reservations, as outlined above.

# Peer review: Referee response for version 1

**DOI:** 10.5256/f1000research.14779.r32403

**Published:** 2018-03-28

**Authors:** Helen Elsey

**Affiliations:** 1 Nuffield Centre for International Health and Development, University of Leeds, Leeds, USA

This is a valuable paper providing in-depth information on pregnancy related complications and emergencies in the context of Bangladesh. It draws on the global and Bangladesh literature and situates the study well within the existing evidence.

 The main concern with the paper the presentation and description of the methods. The study is described  as a 'qualitative case study'. The authors have completed a review of record of women with complications during pregnancy and delivery and present the quantitative findings of this review. They have then randomly selected 15 cases for in-depth interviews. This sounds more like a mixed methods study. Greater clarity on the qualitative interviews is required i.e. how were these conducted, by whom, where, was their an interview guide, did the women consent? The decision to randomly select women needs to be justified; with a mixed methods design, the authors could have purposively selected women from their case notes to explore particular issues in the interviews.

 Greater clarification on how the interviews were analysed: were they audio-recorded and transcribed? how did they come up with the 5 headings in the table - are these the emerging themes from the qualitative analysis?

 While Table 4 is interesting and gives a good insight into the cases, the paper would be greatly strengthened if this descriptive presentation could be synthesises and reported in the results. This synthesis of the key issues emerging from the interviews should also be included in the abstract.

 The quotations provided in Table 4 come from various people, not just the women e.g. TBAs, nurse, panchayat etc. Does this mean these individuals were interviewed? if so details of the methods used for these qualitative interviews also need to be given.

 Further details on the socio-economic situation of the tea gardens would help readers understand the context.

 Acronyms and Bangladeshi-specific words should be spelt out for an international audience.

 This has the potential to be an interesting and valuable paper, but greater clarity on the qualitative methods and analysis is required before being indexed.

I have read this submission. I believe that I have an appropriate level of expertise to confirm that it is of an acceptable scientific standard, however I have significant reservations, as outlined above.
